# Effect of Heat Treatment on Microstructure and Selective Corrosion of LPBF-AlSi10Mg by Means of SKPFM and Exo-Electron Emission

**DOI:** 10.3390/ma14195602

**Published:** 2021-09-27

**Authors:** Marina Cabrini, Sergio Lorenzi, Cristian Testa, Diego Manfredi, Mariangela Lombardi, Alberta Aversa, Francesco Andreatta, Lorenzo Fedrizzi, Yuri Dekhtyar, Hermanis Sorokins, Tommaso Pastore

**Affiliations:** 1 Department of Engineering and Applied Sciences, School of Engineering, University of Bergamo, 24044 Dalmine (BG), Italy; sergio.lorenzi@unibg.it (S.L.); cristian.testa@unibg.it (C.T.); tommaso.pastore@unibg.it (T.P.); 2 Research Unit of Bergamo of National Interuniversity Consortium of Materials Science and Technology (INSTM), 24044 Dalmine (BG), Italy; 3 Research Unit of Bergamo of Center for Colloid and Surface Science (CSGI), 24044 Dalmine (BG), Italy; 4 Department of Applied Science and Technology, Politecnico di Torino, 10129 Torino, Italy; diego.manfredi@polito.it (D.M.); mariangela.lombardi@polito.it (M.L.); alberta.aversa@polito.it (A.A.); 5 Dipartimento Politecnico di Ingegneria e Architettura, Università di Udine, Via delle Scienze 206, 33100 Udine, Italy; francesco.andreatta@uniud.it (F.A.); lorenzo.fedrizzi@uniud.it (L.F.); 6 Biomedical Engineering and Nanotechnologies Institute, Riga Technical University (LV), 1 Kalku Street, 1658 Riga, Latvia; dekhtyar@latnet.lv (Y.D.); hermanis.sorokins@rtu.lv (H.S.)

**Keywords:** AlSi10Mg, laser powder bed fusion (LPBF), exo-electron emission, volta potential, scanning Kelvin probe force microscopy (SKPFM)

## Abstract

The paper deals with the evolution of the microstructure of AlSi10Mg alloy obtained by laser powder bed fusion (LPBF), as a function of the post-processing heat treatment temperature. This was approached by complementary methods including FE-scanning electron microscopy, scanning Kelvin probe force microscopy and exo-electron emission techniques. The fast cooling rate of the LPBF process as compared to traditional casting produces a very fine microstructure with high mechanical properties and corrosion resistance. However, the LPBF-AlSi10Mg alloy can be susceptible to selective corrosion at the edge of the melt pools generated by the laser scan tracks. Post-process thermal treatments of the Al alloy induce a marked modification of the silicon network at melt pool edges, in particular at high temperature such as 400 °C. It was found that this is associated to a more homogeneous distribution of Volta potential. Analysis of exo-electron emission confirms the silicon diffusion during thermal treatment. The modification of the silicon network structure of the LPBF-AlSi10Mg during thermal treatment reduces the susceptibility to selective corrosion.

## 1. Introduction

The good combination of strength, heat conductivity, and low weight make the AlSi10Mg aluminum alloy an optimal choice for several applications in automotive, aerospace and automation [1,2]. Its applications include housings, ductwork, engine parts, production tools and molds [3,4,5].

In the last few years many studies have investigated the effect of heat treatment on the corrosion behavior of AlSi10Mg alloy obtained by means of laser powder bed fusion (LPBF) [6,7], also known with trade names as direct metal laser sintering (DMLS) [8,9,10,11] or selective laser melting (SLM) [10,12,13,14,15]. The corrosion resistance of the LPBF alloys is affected by different factors including the effect of the 3D printed surface [16,17] and that of the microstructure [18,19]. Considering the surface, the LPBF process produces a high surface roughness, and usually defects like emerging porosities [20] or the so called “balling” phenomenon, which is the formation of metallic droplets instead of a uniform spread of liquid metal on the molten surface. Moreover, superficial residual stresses due to the thermal gradients typical of the building process are present in materials by LPBF, with the highest values located at the outer surface. Therefore the complex structure of the as printed surface could impair the nucleation and growth of the native protective oxide film, affecting the passivity and pitting resistance of the material [21,22]. Pickling treatment, shot peening or mechanical polishing [11], and anodizing [23] can improve the corrosion resistance of the 3D printed alloys.

On the other hand, the microstructure of the AlSi10Mg alloy by LPBF is different from that of cast and wrought alloys with the same chemical composition. During the 3D printing, a powder layer is melted and rapidly solidified creating a unique microstructure of Al cellular grains oversaturated with Si and surrounded by Si-microcrystals or the eutectic phase [24]. Considering a section of a sample along a plane parallel to the building direction, it could be stated that the Al grain growth follows a radial direction from the center of the melt pool generated by the laser scan track [14,25]. To complete melt a 2D section of a sample for each layer, the laser follows a path using parallel hatching lines separated by a certain distance. When a second track is partially overlapped to a solidified one, a partial fusion of the first track border occurs, whereas the near zone is thermally altered, and it can be labelled as the edge of the melt pool. Different microstructures in the center, and at the edge of the melt pools are created depending on printing parameters, including the laser power, scanning speed and hatching strategy [20,21,26,27].

AlSi10Mg alloy obtained by LPBF generally shows higher corrosion resistance than the cast counterpart [9,10]. However, a unique morphology of corrosion with preferential dissolution of the Al matrix surrounding secondary particles of primary Si is observed [7,12,13,28]. This can lead to the selective attack of the edge of the melt pool, which is often reported in literature [20,29]. The selective dissolution of the edge of the melt pool was attributed to the coarsening of Si particles, which renders discontinuous the shielding network of Si at the melt pool edges [11]. Revilla et al. [15] found higher differences between the Volta potential of the Si phase and the Al matrix at the edge of the melt pool with respect to the center of the melt pool, for the alloy in both as built condition and after a stress-relieving treatment at 300 °C for 2 h [13,22].

The different microstructures of border and edge of the melt pools are strongly dependent on main process parameters as stated before, including building platform temperature, and post-processing heat treatments, like stress relieving [7,13,28,30]. In a previous study, it was found that the melt pool tracks of AlSi10Mg by LPBF with a laser power of 195 W, no longer become visible through optical microscopy after a thermal treatment at 400 °C for 2 h [7,31]. As reported by Thijs et al. [25], during a post-process heat treatment, the Si is rejected from the aluminium matrix, forming microcrystals uniformly distributed on aluminium dendrites. These Si crystals form a network that is interrupted in the correspondence of the edge of the melt pool, where the Si is present as isolated particles. This leads to selective attack at the edge of melt pools, which is observed after post-process thermal treatments at 200 °C and at 300 °C, while it is not observed at 400 °C, or even at higher temperature, due to the coalescence of the Si phase and consequent destruction of the melt pool macrostructure.

Similar effects were found by other authors for heat treatments at different temperatures, on samples produced with different systems and, therefore, the main process parameters. Rubben et al. [13] observed similar electrochemical behavior in a 0.1 M NaCl solution, for the AlSi10Mg alloy by LPBF built with a laser power of 500 W, after an artificial aging at 170 °C for 6 h, and stress relieving at 300 °C for 2 h and another at 250 °C for 2 h. However, the corrosion morphology of the samples changed noticeably from a superficial corrosion attack coupled with formation of micro-cracks along the melt pool edges for the as built alloy and for that aged at 170 °C, to a more severe penetrating corrosion attack still preferentially oriented along the edge of the melt pool after stress-relieving treatments. According to Rubben et al. [13], long-term exposure in the electrolyte seemed to cause more severe degradation for the alloy subjected to thermal treatment at 250 °C and at 300 °C as compared to the as built alloy [13]. Rafieazad et al. [28] investigated the effect of the microstructure of heat-treated AlSi10Mg alloy built with a laser power of 360 W. They found that the corrosion morphology changes from a penetrating selective attack along the melt pool edges in the as built alloy and in that heat-treated at 200 °C to a more localized corrosion of the α-Al matrix along the border of Si particles when the alloy was heat-treated at 300 °C. This modification of the attack morphology was attributed to the more uniform microstructure obtained at 300 °C.

It can be stated that the laser power affects the thermal gradient and, therefore, the cooling rate of the alloy; consequently, it affects the microstructure of as built specimens and therefore the “starting condition” for the post-process thermal treatment. However, the correlation between LPBF AlSi10Mg alloy microstructure, heat treatment and the corrosion behavior has not been completely clarified.

In addition to the aspects discussed above, the presence of Mg in the alloy composition suggests the possibility to induce age hardening through Mg_2_Si precipitation [20,28,32]. The Mg_2_Si precipitates could greatly affect the corrosion behavior of Al-Si-Mg alloys [33,34]. Literature data are controversial on the possibility of precipitation of Mg_2_Si particles during 3D printing and/or post-processing heat treatments. Moreover, the effect of these second phase particles on the corrosion behavior of the alloy is still not clarified. Marola et al. [33] reported differential scanning calorimetry (DSC) thermograms for AlSi10Mg alloy printed with a laser power of 195 W and a building platform temperature of 100 °C. They attributed exothermic peaks at 317 °C to the Mg_2_Si precipitation and those at about 340 °C to possible formation of Fe-containing intermetallics [35]. In another study, M. Rafieazad, et al. [11] reported DSC thermograms showing two exothermic peaks at around 233 °C and 273 °C for the same alloy but printed with a laser power of 360 W and a building platform temperature of 200 °C. The first peak was attributed to the precipitation of Mg_2_Si in β” form while the second peak was correlated to the activation of Si interdiffusion in the Al matrix. Si interdiffusion controls the precipitation, coarsening, and spheroidization of Si particles in the alloy [28]. Moreover, Rafieazad et al. reported that heat treatment at 233 °C can promote the precipitation hardening of the alloy through formation of the Mg_2_Si β phase. On the other hand, increasing the heat-treatment temperature from 200 °C to 350 °C, resulted in a drastic decrease of the micro-hardness due to the increase of size and spheroidization of the Si particles [28]. Therefore, the effect of precipitation hardening is not completely clear from this study. The same authors observed Si phase precipitation via solid-state diffusion at around 273 °C.

Fiocchi et al. detected two exothermic transformations by DSC in a LPBF AlSi10Mg alloy with 300 W laser power and a building platform at 25 °C. They found an exothermic peak at 263 °C and another at 294 °C. They correlated the first peak to Mg_2_Si precipitation, and the second peak to the rupture of the Si network and spheroidization of the particles [34].

Gu et al. reported the presence of Mg_2_Si in LPBF specimens with a laser power of 200 W, after heat treatments at 300 °C and 400 °C [36]. Revilla et al. reviewed the corrosion behavior of additively manufactured (AM) Al alloys. They reported that the actual formation of Mg_2_Si particles in AM Al–Si alloys has not yet been confirmed, due possibly to the extremely high cooling and solidification rates resulting in a very fine (out-of-equilibrium) distribution of alloying elements with almost no time for the formation of the usual precipitates [20].

In the present work, the effect of the post processing heat treatment on the microstructure of LPBF AlSi10Mg alloy was investigated combining exo-electron emission (EEE) and scanning Kelvin probe force microscopy (SKPFM). These methods provide complementary information on the electron extraction work function of the material. The EEE method is based on the non-stationary electron emission that takes place at energy levels lower than the work function. The emission from solids can originate from various external excitations such as mechanical deformation, chemisorption, X-ray irradiation, electron bombardment and phase transitions [37], which cannot be explained within the framework of the classical photoelectric effect, thermionic emission, secondary electron emission or other well-known processes [38]. EEE is basically a surface phenomenon, typically provided at the mm scale. It measures the change in surface potential or electron work function [39] and it is extremely sensitive to the surface condition of the sample. In particular, it can be expected that thermal treatment can affect the work function since it can restructure/modify the surface of as printed LPBF materials. T. Górecki and C. Górecki reported the possibility to observe exo-electron emission due to phase changes and precipitation of second phases if the specimen is stimulated with light of appropriate wavelength [40,41,42]. Sujak and co-workers investigated by EEE the early stages of natural aging of the industrial AW-6082 after solution heat treatment at 537 °C and quenching in ice water [42]. The curve of the EEE intensity decay exhibited a peak corresponding to the maximum rate of Mg_2_Si precipitation. They observed that decay for the EEE accompanying the precipitation of the Mg_2_Si phase can be attributed to the annihilation of the crystal lattice defects created during the quenching procedure [40]. A similar situation could be supposed for the LPBF process, in which the high cooling rate during melt pool solidification creates a Si and Mg supersaturation in the α-Al matrix, hindering the precipitation of Mg_2_Si. Therefore, the EEE method could evidence the microstructure modification in the alloy during heating. In this work, the EEE method was employed in order to evaluate possible precipitation of Mg_2_Si particles and to obtain information about coarsening of the Si phase during post processing of LPBF AlSi10Mg alloy. Moreover, this method was combined with the use of SKPFM, which is a local technique to measure the Volta potential at the micro/nano scale. The Volta potential is related to the energy to remove electrons from the material Fermi level of a surface [15,43]. In the present work the SKPFM was used to study the Volta potential distribution between the center and the edge of the melt pool in order to correlate the structural modifications of the alloy induced by different post-processing treatments to its corrosion behavior.

## 2. Materials and Methods

### 2.1. Materials and Specimens

The specimens were prepared using gas-atomized AlSi10Mg alloy powder supplied by EOS GmbH (Electro-Optical Systems, Krailling, Germany) with the nominal chemical composition shown in Table 1. The detailed description of the LPBF process for the production of the specimens is given in previous works [44,45]. The specimens’ laser power was 195 W, the scanning speed 800 mm/s, the hatching distance 0.17 mm, the layer thickness 0.03 mm, and the temperature of the building platform 35 °C; a rotation of the scanning direction of 67° between consecutive layers was performed in order to achieve optimal overlapping of the laser tracks. Disk specimens 15 mm in diameter and 5 mm in height were built with the circular surface perpendicular to the building platform (XZ direction). The as-built specimens without any further heat treatment are named un-treated (UT). Part of the as-built specimens were heat-treated for 2 h at 300 °C (2h-300) or for 2 h at 400 °C (2h-400), and then cooled in air.

### 2.2. Optical and Field-Emission Scanning Electron Microscopy/Energy-Dispersive X-ray Spectroscopy (FESEM/EDS) Analysis

The microstructure of the specimens manufactured by LPBF was investigated by optical microscopy (Nikon Europe BV, Amsterdam, Netherlands) and field-emission scanning electron microscopy (FESEM JSM-7610FPlus, Zeiss, Oberkochen, Germany). The samples were grinded with abrasive papers until 4000 grit, then polished with an aqueous suspension of 0.3 μm colloidal alumina. The specimens were investigated in the polished condition without etching in order to avoid any modification of the original microstructure of untreated and heat-treated specimens and after metallographic attack using Keller’s reagent (5 mL HNO_3_ 69.5%, 3 mL HCl 36%, 2 mL HF 50% in 190 mL of distilled H_2_O for 15 s at 23 °C). Elemental map analysis was also performed on samples without etching using the energy-dispersive spectroscopy (EDS) detector of the FESEM (Oxford, High Wycombe, UK). At least 5 elemental maps were acquired for each sample investigated (UT, 2h-300 and 2h-400). Similarly, about 10 EDXS spectra were acquired to evaluate the chemical composition of the intermetallics.

### 2.3. Exo-Electron Emission (EEE)

The EEE measurements were carried out using an exo-electron spectrometer in vacuum 10^−3^ Pa on AlSi10Mg specimens printed with a building platform temperature of 100 °C. During measurements, the samples were heated from room temperature to 550 °C with a heating rate of 10 °C/min. The emitted electrons were detected using a secondary electron multiplier SEM-6M (VTC Baspik, Republic North Ossetia-Alania, Russian Federation) as reported in [46].

### 2.4. Scanning Kelvin Probe Force Microscope (SKPFM)

Topographic maps of LPBF etched samples were acquired with a with a Nanoscope III multimode atomic force microscope (AFM) equipped with an Extender TM electronic module (Bruker, Billerica, Massachusetts, USA). The SKPFM was employed to obtain surface potential (Volta potential) maps of the LPBF specimens. These maps were obtained on polished samples without etching in order to avoid topographic artefacts. The topographic and Volta potential maps were acquired using n^+^—silicon tips coated with PtIr_5_ at room temperature with a relative humidity of 40–65%. The scan frequency was 0.1 Hz and the scan height in lift mode was 100 nm.

## 3. Results and Discussion

**Figure 1:**
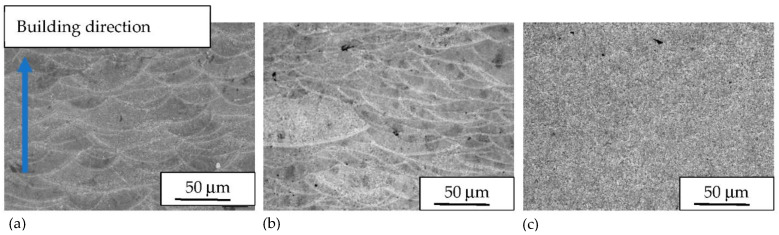
Optical images of AlSi10Mg after etching of (**a**) UT, (**b**) 2h-300 and (**c**) 2h-400 specimens. (The images are referred to the direction parallel to the building direction—XZ).

The macrostructure of the UT samples with cross section parallel to the building direction (XZ) shows semi-circular shaped melt pools, partially overlapped on previous tracks (Figure 1a). The macrostructure of the specimens does not change after heat treatment at 300 °C and the melt pools are still visible on the sample surface (Figure 1b). The edges of the melt pools cannot be distinguished well when the heat treatment temperature is increased to 400 °C (Figure 1c).

**Figure 2:**
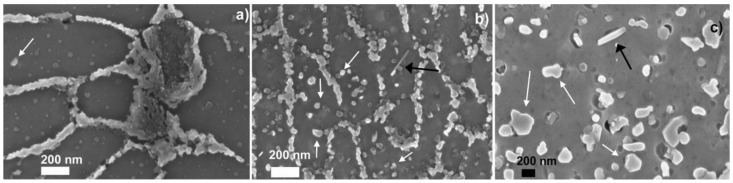
Field-emission scanning electron microscopy (FESEM) images at high magnifications of AlSi10Mg microstructures after etching of (**a**) UT (**b**) 2h-300 and (**c**) 2h-400 specimens. Examples of Si particles are indicated by the white arrows, while the Fe rich needle like particles are indicated by black arrows. (The images are referred to the direction parallel to the building direction—XZ).

Figure 2 reports the microstructure at high magnification of the alloy with different heat treatments. The UT alloy exhibits α-Al phase with Si as round particles or eutectic phase along the Al grains (Figure 2a). Some small Si crystals are visible inside the Al matrix. The heat treatment at 300 °C induces a modification of primary Si mainly associated to an increase of particle density (Figure 2b). However, the size of the primary Si remains noticeably small after heat treatment at this temperature. Moreover, it can be seen that the number and size of isolated Si particles in the α-Al phase grains is higher than in the UT alloy. Some Fe rich needle-like particles are visible inside the Al matrix (Figure 2b). Heat treatment at 400 °C induces a marked coalescence of the primary Si particles, which is associated to the formation of relatively large round second phases particles (Figure 2c). As highlighted by optical images (Figure 1c) the structure of the melt pool boundaries cannot be recognized even at high magnification.

FESEM analysis could not highlight the presence of Mg_2_Si precipitates on as-built or heat-treated specimens. Therefore, phase modifications were investigated using the EEE technique.

**Figure 3:**
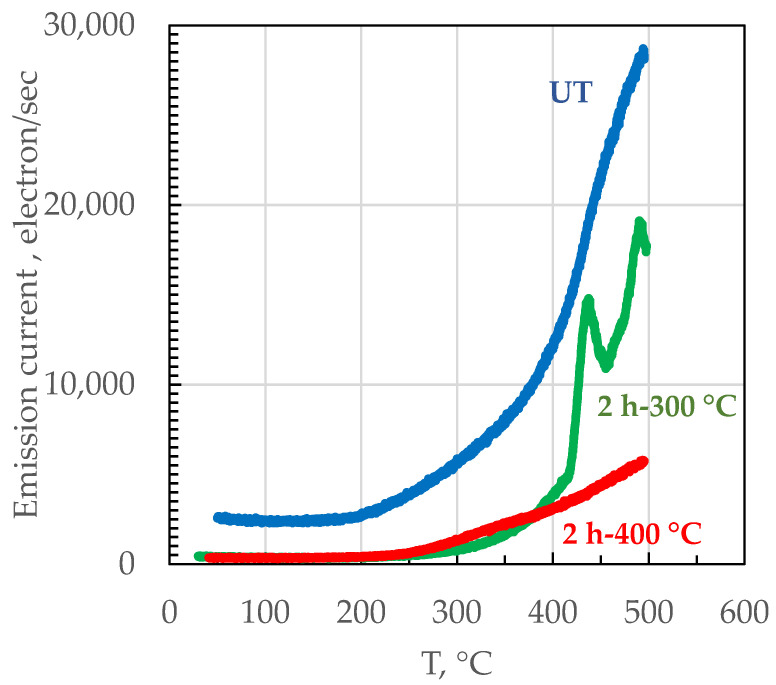
Exo-electron emission (EEE) of the AlSi10Mg LPBF specimens after different post heat treatments as a function of temperature (XZ-specimens).

Figure 3 shows the exo-electron emission current in the temperature range from 20 °C to 500 °C for the UT, 2h-300 and 2h-400 specimens. The emission current of the UT condition is higher than for the 2h-300 and 2h-400 specimens in the entire temperature range. This could be attributed to the high lattice distortion of the UT specimens, which is generated by fast cooling during the 3D manufacturing process resulting in a marked oversaturation with Si of the Al matrix. The UT alloy exhibits a rather sharp increase of the emission current above 200 °C. However, no emission peaks could be identified between 200 °C and 300 °C, which is the temperature range for precipitation of Mg_2_Si particles during aging. The emission current of the 2h-300 sample exhibits a progressive increase above 250 °C with an emission peak at about 420 °C. The 2h-400 sample displays low emission current relative to other samples and the peak observed at 420 °C for the 2h-300 condition is not observed after post-processing at 400 °C.

The absence of emission peaks in the range 200–300 °C for the UT and post-processed samples seems to indicate that precipitation of Mg_2_Si particles does not occur for the LPBF AlSi10Mg alloy investigated in this work. Moreover, it is unlikely that the emission peak at 420 °C is due to precipitation of Mg_2_Si particles in the 2h-300 sample because the peak temperature is significantly higher than that at which precipitation is observed by DSC for this alloy [35]. The emission peak at 420 °C for the 2h-300 sample might be related to the coalescence of the primary Si particles, which is observed after post-processing thermal treatment at 400 °C (Figure 2c). Indeed, the 2h-300 sample does not show significant coalescence of the Si phase in Figure 2b. It can be expected that this process could happen at temperatures above 400 °C during the acquisition of the EEE spectrum. This is further supported by the absence of the peak at 420 °C in the 2h-400 sample since a significant coalescence of the Si phase took place during the post-processing thermal treatment of the alloy, as visible in Figure 2c. Considering that in the same range of temperature it is possible to observe a sharp increase of the EEE of the UT specimens, but not for the 2h-400 °C ones, it could be stated that this enhancement is due to the coalescence of Si crystal to rounded particles. This effect is less pronounced in 2h-400 °C samples because the spheroidization of Si has taken place during the heat treatment. The possible precipitation of Mg_2_Si could be evidenced by the increases of EEE around 200 °C, evident only for the UT specimens, but, on the other hand, the absence of a distinct peak suggests a phenomenon that begins but it does not run out at 200 °C. Moreover, the EEE of LPBF samples appears to be controlled by release of Si from the supersaturated α-Al matrix to form idiomorphic crystals of increasing size during post-processing, in line with FESEM micrographs presented in Figure 2. Relief of residual stresses can contribute to electron emission, in particular for the UT sample. The EEE data are in agreement with the work previously reported by Marola et al. in which the precipitation of Mg_2_Si was not observed in LPBF AlSi10Mg alloy obtained with the same equipment and processing parameters [25]. During the heating of the LPBF alloy, there are two main microstructural modifications: the first is the rejection of silicon present in over-saturated solution in the a-Al matrix with successive coalescence into idiomorphic crystals; the second is the coalescence of micro-dendrites of α-Al into crystals of larger size. This latter phenomenon makes melt-pools disappear. The two processes take place simultaneously in the UT specimens, giving a high and continued increasing EEE with temperature. By contrast, the silicon is fully separated from the aluminum matrix in the 2h-400 °C specimen, that shows only a weak EEE emission starting from 300 °C, which can be attributed to the partial re-crystallization of α-Al. The 2h-300 °C specimens show an intermediate behavior: until 400 °C the curve is practically overlapped with the one of the 2h-400 °C specimens, but increasing the temperature the silicon particles’ coalescence determine the peak at 420 °C. The coalescence of silicon particles separated during the heat treatment at 300 °C probably determines a relaxation of the lattice and a successive partial decreasing of the EEE emission. After this relaxation, the EEE of 2h-300 °C increases again for the coalescence of Al dendrites, as observed in UT specimens. The EEE tests were stopped at 500 °C, while the full annealing of the alloy takes place at higher temperature, so it was not possible to determine if the EEE emission would continue or stop to increase when the aluminium recrystallization is completed. This aspect will be the subject of further research.

**Figure 4:**
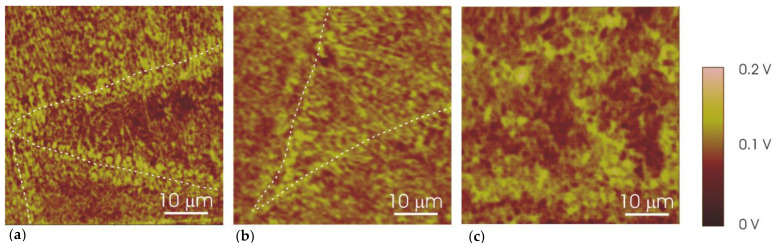
Scanning Kelvin probe force microscope (SKPFM) Volta potential maps for (**a**) UT, (**b**) 2h-300 and (**c**) 2h-400 specimens. The melt pool borders in the UT and 2h-300 specimens are highlighted with dashed lines. (XZ specimens).

The SKPFM Volta potential maps of the UT and thermally treated specimens are shown in Figure 4. The Si phase displays higher potential than the α-Al phase in Volta potential maps. The melt pool borders can be clearly identified in the UT sample (Figure 4a) revealing a continuous Si phase along the borders in line with the microstructure shown in Figure 1a. The Volta potential map of the 2h-300 specimen (Figure 4b) appears similar to that of the UT alloy although the thermal treatment leads to a more homogeneous distribution of the Volta potential between edge and center of the melt pools. In particular, it can be seen that the potential difference between the Si and α-Al phases appears less marked at the melt pool edges for the 2h-300 specimen. The potential map for the 2h-400 specimen (Figure 4c) displays larger Si phase relative to UT and 2h-300 specimens. Moreover, the melt pool edges cannot be identified in the Volta potential map confirming the evolution of the microstructure due to thermal treatment at 400 °C.

**Figure 5:**
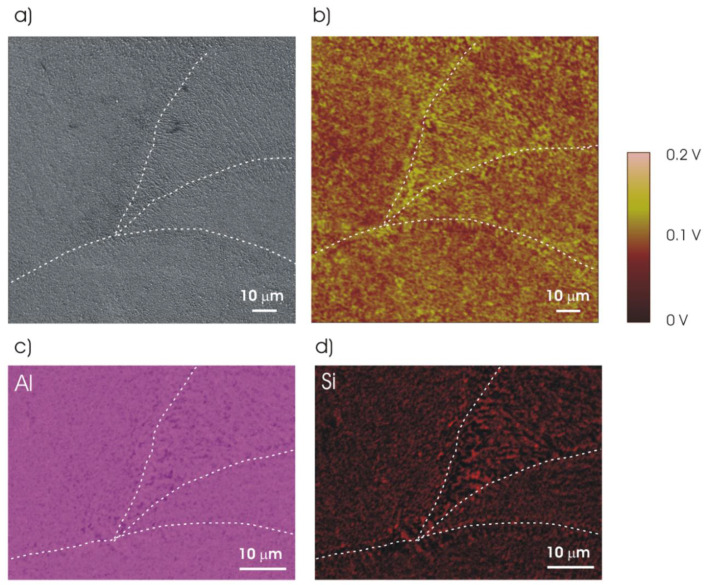
(**a**) Scanning electron microscopy (SEM) micrograph without metallographic attack, (**b**) SKPFM Volta potential map of the same region shown in the SEM micrograph and (**c**,**d**) energy-dispersive X-ray spectroscopy (EDS) elemental maps of Al and Si of the 2h-300 specimen. The melt pool edges are highlighted with dashed lines. (XZ specimens).

**Figure 6:**
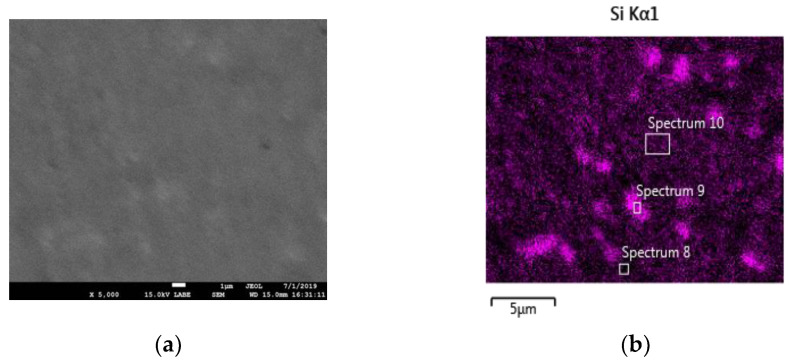
(**a**) SEM image without metallographic attack and (**b**) EDS Si map of the 2h-400 specimen. (XZ specimens).

Figure 5a shows a SEM micrograph of the surface of a 2h-300 specimen and Figure 5b displays the Volta potential map of the same region. Figure 5c,d show the EDS elemental maps of Al and Si, respectively. The specimen was investigated without etching in order to avoid modification of surface potential. As introduced above, the contrast in the Volta potential map is due to the Si phase that exhibits higher potential than the α-Al phase. This is confirmed by the elemental maps of Si and Al, which clearly indicate that a potential contrast is mainly associated with the formation of a Si phase. The higher potential of the Si phase is associated with the more noble behavior of Si relative to the surrounding Al matrix (α-Al phase). Conversely, the Si particles coalesce during the heat treatment at 400 °C, as shown in the EDS map in Figure 6. The size of these particles is almost too small to undertake EDS quantitative analysis. However, Table 2 reports the composition of three different regions of the sample 2h-400 shown in Figure 6b clearly highlighting the presence of a phase (spectrum 9) with a higher Si content than the matrix (spectrum 8 and spectrum 9). Furthermore, there is no evidence of Mg_2_Si precipitation.

Table 3 reports the mean value and standard deviation of potential differences measured between the Si and α-Al phases in order to better evaluate the modification of potential contrast displayed in Figure 4 for specimens with and without thermal treatment. Moreover, potential differences detected at the border and center of the melt pools are given in the table for the specimens in which melt pool edges can be recognized in SEM micrographs and Volta potential maps. The UT specimen displays an average potential difference of 133 mV between the Si and α-Al phases at the edge of the melt pools while this difference is 88 mV in the central region of the pool. This suggests a higher reactivity of the borders relative to the melt pool center. A similar result is reported by Revilla et al. [15]. This trend is confirmed by the 2h-300 specimen. In particular, the potential difference remains rather high (94 mV) at the melt pool edges while it is lower in the central regions (60 mV). The modification of potential differences in the 2h-300 specimen is probably the result of the thermal treatment, which affects the Si phase (Figure 2) and, most likely, induces a redistribution of the alloying elements (Si and probably Mg) in the α-Al phase. The average potential difference between the Si and α-Al phases is 86 mV in the 2h-400 specimen. This is very similar to that observed at the melt pool edges of the 2h-300 specimen. However, the heat treatment at 400 °C makes the distribution of the surface potential more homogeneous than for the UT and 2h-300 specimens. In particular, the marked potential difference between the edges of the melt pool and the internal regions tends to disappear in the 2h-400 specimen, indicating a lower susceptibility to selective attack.

**Figure 7:**
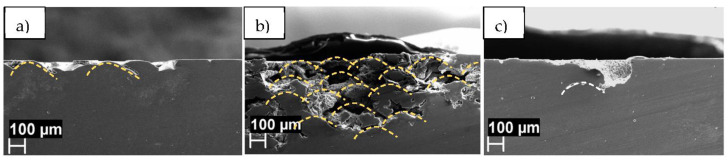
Morphology of corrosion after ISO 11846 test on AlSi10Mg LPBF samples (**a**) UT; (**b**) 2h-300; (**c**) 2h-400: the yellow dotted line underlined the edge of the melt pool that is preferentially corroded; the white dotted line in (**c**) evidences a porosity in the correspondence of the prior edge of the melt pool (after the heat treatment the melt pools disappeared). (Exposed area XY, section XZ).

SKPFM data are in agreement with the corrosion behavior previously reported for the AlSi10Mg manufactured by LPBF [6,7] UT and 2h-300 specimens are susceptible to selective attack along the melt pool boundaries, as showed by the yellow dotted line in Figure 7; the white dotted line in (Figure 7c) evidences a porosity in the correspondence of the prior edge of the melt pool, whereas the melt pools disappeared after the heat treatment. The selective attack of the edge of the melt pool is promoted by galvanic coupling due to the high Volta potential difference between the Si and α-Al phases [7]. In contrast, thermal treatment at 400 °C leads to a more uniform microstructure, which exhibits pitting attack uniformly distributed on the exposed surface in electrochemical tests [7]. The Si phase is a preferential site for pitting because a relatively high potential difference exists between this phase and the α-Al phase even after thermal treatment at 400 °C. Previous work highlighted also that the 2h-300 specimen is more prone to selective attack of the melt pool edges than the UT specimen. The continuous Si phase network observed at the melt pool edges in the UT sample (Figure 2a) tends to evolve into a discontinuous Si phase in the 2h-300 specimen (Figure 2b). This, in combination with the existence of rather high potential differences between Si and α-Al phase, could explain the high susceptibility of the 2h-300 specimen to selective attack at the melt pool edges.

It is likely that the modification of the melt pool structure and chemical composition due to thermal treatments at 300 °C and at 400 °C involves redistribution of alloying elements (Si and Mg) and most likely, the evolution of the microstructure of the UT sample into a structure like that of the alloy produced with conventional manufacturing processes. This could also involve the precipitation of Mg_2_Si phase, although this is not well documented for the AlSi10Mg produced by LPBF nor observed in this work.

## 4. Conclusions

The effect of post-processing heat treatment on the microstructure evolution and on the consequent corrosion resistance of the AlSi10Mg alloy by LPBF was evaluated by means of EEE and SKPFM techniques.

Heat treatments at 300 °C and at 400 °C modified the distribution of the primary Si, which is present as a nearly continuous network along melt pool edges in the as-built alloy. In particular, post-processing at 400 °C led to a marked coalescence of the primary Si above 400 °C, which was confirmed by EEE measurements. The EEE could not evidence Mg_2_Si precipitation in the LPBF alloy.

The SKPFM technique highlighted the Volta potential differences between the Al and Si, which are higher at the borders of melt pools. The post-processing heat treatment at 400 °C for 2 h led to a more homogeneous distribution of Volta potential and an overall decrease of potential difference between primary Si and α-Al matrix. High Volta potential differences at the melt pool borders are the main cause of the selective attack observed in the as-built condition and after post processing at 300 °C. The susceptibility to selective attack of the melt pool borders decreased with a thermal treatment at 400 °C due to coalescence of the Si primary phase.

## Figures and Tables

**Table 1:** Nominal chemical composition of the AlSi10Mg powder for the laser powder bed fusion (LPBF) process.

Element	Si	Fe	Cu	Mn	Mg	Ni	Zn	Ti	Al
% weight	9–11	≤0.55	≤0.05	≤0.45	0.2–0.45	≤0.05	≤0.1	≤0.15	bulk

**Table 2:** Quantitative EDS analysis of the spectra in Figure 6b.

Spectrum Label	Spectrum 8	Spectrum 9	Spectrum 10
Mg	0.3	0.5	0.4
Al	93.2	58.5	89.5
Si	5.5	40.5	10.1
Fe	-	0.5	-

**Table 3:** Volta potential difference between Si and α-Al phases in AlSi10Mg LPBF samples after different post-heat treatments.

Specimen	Edge of the Melt Pool [mV]	Centre of the Melt Pool [mV]
UT	133 ± 9	88 ± 6
2h-300	94 ± 8	60 ± 6
2h-400	86 ± 10	

## Data Availability

Not applicable.
